# Perspectives of Spanish-Speaking Caregivers on Pediatric Patient Portal Use

**DOI:** 10.1055/a-2688-3992

**Published:** 2025-10-01

**Authors:** Gabriel Tse, Stephanie Squires, Katherine Hu, Michelle M. Kelly, Bonnie Halpern-Felsher, Jennifer Carlson

**Affiliations:** 1 Division of Pediatric Hospital Medicine, Department of Pediatrics, Stanford School of Medicine, Palo Alto, California, United States; 2 Division of Pediatric Hospital Medicine & Complex Care, Department of Pediatrics, University of Wisconsin School of Medicine and Public Health, Madison, Wisconsin, United States; 3 Division of Adolescent Medicine, Department of Pediatrics, Stanford School of Medicine, Palo Alto, California, United States

**Keywords:** patient portals, digital health, language equity

## Abstract

**Objectives:**

Disparities exist in patient portal use among non-English-speaking caregivers of pediatric patients. This study aims to evaluate the reasons behind Spanish-speaking caregivers' use of patient portals and identify facilitators and barriers, focusing on those caring for children with chronic conditions.

**Methods:**

We conducted semi-structured interviews and surveys with Spanish-speaking caregivers of pediatric patients with chronic conditions at an academic pediatric health network in California. Data were transcribed, coded, and analyzed using inductive thematic analysis.

**Results:**

Twenty caregivers participated. Participants primarily accessed patient portals via their smartphones, and most accessed the patient portal at least weekly. Three main themes emerged: perceived benefits (managing appointments, medications, and results), facilitators that improved use (support from healthcare professionals), and barriers that negatively affected use (differences in language, health literacy, and digital health literacy).

**Conclusion:**

Spanish-speaking caregivers find patient portals beneficial but face significant barriers related to language discordance and differences in health literacy and digital health literacy. This study highlights the need for health systems to provide language concordance within patient portals and consider innovative solutions that promote equitable use.

## Background and Significance

Patient portals are a type of digital health technology that engage patients with their healthcare through functions such as accessing their personal health records, making appointments, and sending messages to their healthcare team via a website or mobile application. 1 Studies have described the benefits of patient portal use in both adult and pediatric patients, including improved medication adherence, patient-provider relationships, and health knowledge. 1 2 3 Adult patients with a preferred language other than English (LOE) report greater benefit from clinical notes shared through patient portals compared with their English-speaking counterparts, and they are more likely to report that reading notes made them feel more in control of their care and helped them remember their medical plan. 4 5

Despite these benefits, significant disparities in patient portal use exist, with patients with LOE having lower enrollment rates compared with English-speaking patients, despite similar levels of interest between these groups. 6 7 8 Disparities in patient portal use may lead to intervention-generated inequalities, where the implementation of digital health technology worsens, rather than improves, already existing health disparities between these two populations. 6 9 Adult data indicate that patients with LOE have lower rates of patient portal use due to various factors, including limited digital health literacy, which is defined as the ability to seek, find, understand, and appraise health information from electronic sources. 10 11 12 As healthcare becomes increasingly digitized, digital health literacy is essential for navigating and participating in one's healthcare and has been referred to as a “super social determinant of health.” 13 Furthermore, it is positively correlated with health literacy. 14 However, while these factors have been documented in adult studies, there is limited understanding of how caregivers of pediatric patients with LOE (LOE caregivers) use patient portals and the barriers that they encounter. This gap in knowledge could contribute to further disparities in patient portal use between adult and pediatric populations. 6

While multiple studies have focused on improving patient portal enrollment in LOE populations, few studies have gathered the perspectives of those already using patient portals. Understanding these perspectives is crucial to guiding strategies to improve use in LOE caregivers, as caregivers are often the primary users of patient portals in pediatrics. Thus, the aims of this study are to evaluate why LOE caregivers, specifically Spanish-speaking caregivers, use patient portals and identify facilitators and barriers that influence their use. We will focus on caregivers of patients with chronic conditions as they have higher patient portal utilization and may have more insights about using patient portals, compared with otherwise healthy patients. 15 16 17 18 Spanish-speaking caregivers were chosen because Spanish is the most common non-English language spoken in the United States and at our institution, Spanish-speaking patients have significantly lower patient portal enrollment and usage rates compared with other LOE patients. 19

## Methods

### Study Design

This qualitative study was conducted at Stanford Medicine Children's Health, an academic pediatric health network affiliated with Stanford University School of Medicine. Our institution uses MyChart (Epic Systems, Verona, Wisconsin, United States) as its patient portal platform, which has functionalities that include viewing and scheduling appointments, reading clinical notes, accessing results, and messaging care teams. 20 MyChart has both English and Spanish versions, although unstructured data (e.g., free text, clinical notes, imaging reports, and medications) are not automatically translated. We conducted semi-structured interviews and surveys of parents or legal guardians (hereafter “caregivers”) of pediatric patients with the following eligibility criteria:

–Caregiver's preferred language is other than English, and the caregiver identifies as Spanish speaking.
–Patient of the caregiver is less than 12 years old, as proxy access to the patient portal changes after a patient turns 12.
–Patient has an activated proxy patient portal account that the caregiver can access.
–Patient has at least one chronic condition. 17

Eligible participants were identified in multiple pediatric subspecialty clinics through the electronic health record (allergy, complex care, gastroenterology, neurology, and rheumatology clinics). Convenience sampling was used for recruitment. Caregivers were approached by a member of the research team with a certified medical interpreter about the purpose of the study and invited to participate. Participants were approached in a private clinic room either before or after their provider had seen them. After providing written consent, a member of the research team would call to schedule the interview. Sample size was determined based on when thematic sufficiency was reached. 21

A bilingual research member trained in qualitative interviewing conducted the semi-structured interviews via telephone. Participants also completed a survey in Spanish through Qualtrics (Qualtrics, Provo, Utah, United States) on their own devices. Each caregiver was provided a $50 gift card for their participation. The manuscript follows COREQ guidelines for transparent reporting of qualitative research. 22 This study was approved by Stanford's Institutional Review Board.

## Interview Guide and Survey Development

An interview guide was developed based on the aims of the study and after performing a literature review of qualitative studies exploring patient portal use. 7 23 24 The Technology Acceptance Model was used as a conceptual framework to inform the development of the interview guide questions. 25 There were two sections of interview questions that reflected the two factors that influence technology use, based on the Technology Acceptance Model: perceived usefulness (i.e., why caregivers use patient portals) and perceived ease of use (i.e., perceived barriers and facilitators to patient portal use). Questions were revised iteratively by the study team until agreement was reached. The interview guide was reviewed with our institution's Latinx Family Advisory Council and piloted and revised for clarity with three Spanish-speaking caregivers prior to implementation.

The survey was developed to gather sociodemographic data and better characterize participants' access to technology, patient portal use, and perceived digital health literacy. Survey questions were either developed by the research team or adapted from previous published surveys that gather data on patient portal use. 26 A three-point validated digital health care literacy questionnaire was used to assess perceived digital health literacy ( Supplementary Appendix A >, available in the online version only). 27 The survey was translated into Spanish by a certified translator and completed anonymously.

### Data Analysis

The interviews were audio recorded, transcribed verbatim, and translated into English by a certified Spanish translator. The transcript data were deidentified and uploaded to Dedoose (version 9.2.006, SocioCultural Research Consultants, Los Angeles, California, United States) for qualitative data management. Inductive thematic analysis was used to identify, gather, and analyze themes within the data. 28 Two researchers (GT and SS) trained in qualitative methods independently reviewed each transcript and developed codes a priori. The two researchers met iteratively after every fifth transcript to develop the codebook and adjudicate differences in codes. Once a final codebook was created, the two researchers applied the codebook to independently code all transcripts. Codes were organized into categories, which were then used to identify potential themes and subthemes. These themes were reviewed and refined by three further reviewers (MMK, BHF, and J.C.). Throughout data coding and analysis, the research team engaged in reflexivity to critically appraise how subjective assumptions and biases influenced the research process. 29

## Results

A total of 20 caregivers participated in this study. All participants were mothers. The median age was 33.5 years old (25–47), most (75%) did not complete high school, and half (50%) described their financial situation as not meeting or just meeting basic expenses with nothing left over ( Table 1 ).

**Table 1:** Participant-reported characteristics, *n* = 20

Characteristic	*n* (%)
Age, y, mean (SD)	35 (7.4)
Education level	
Less than high school	15 (75)
Completed high school	3 (15)
Some college completed	1 (5)
Completed college or higher	1 (5)
Financial situation	
Does not meet basic expenses	3 (15)
Just meets basic expenses	7 (35)
Meets basic expenses with a little left over	8 (40)
Lives comfortably	1 (5)
Prefer not to say	1 (5)
Ability to speak English	
Very well	0
Well	2 (10)
Not well	9 (45)
Not at all	9 (45)
Ability to read English	
Very well	0
Well	3 (15)
Not well	9 (45)
Not at all	8 (40)
Digital health care literacy scale a (SD)	9.0 (2.26) [range: 5–12]

a Digital healthcare literacy scale has a score of 0 to 12, with higher scores representing higher degrees of digital healthcare literacy.

In terms of technology access, 85% had reliable internet access at home. Most participants (95%) had access to a smartphone, and fewer (55%) had access to a computer or tablet. All participants usually accessed the internet via their smartphones, with 15% also using a tablet. Most (75%) accessed their child's patient portal at least once weekly ( Table 2 ).

**Table 2:** Participant-reported technology access and patient portal use, *n* = 20

	*n* (%)
Do you have reliable internet access?	
Yes	17 (85)
No	3 (15)
Do you have access to a smartphone?	
Yes	19 (95)
No	1 (5)
Do you have access to a computer/tablet?	
Yes	11 (55)
No	9 (45)
How do you usually access the internet? (Select all that apply)	
Smartphone	20 (100)
Tablet	3 (15)
How often do you access your child's patient portal?	
More than once per week	10 (50)
Once per week	5 (25)
A few times per month	3 (15)
Once a month	1 (5)
Once every few months	0
A few times per year	1 (5)
How difficult is it for you to use the patient portal?	
Very difficult	0
Difficult	3 (15)
Neutral	4 (20)
Easy	9 (45)
Very easy	4 (20)
How do you translate material in the patient portal? (Select all that apply)	
Ask your child	5 (25)
Ask a family member	3 (15)
Ask a friend	1 (5)
Use an online translation tool	13 (65)

Three overarching themes were identified, including perceived benefits, facilitators, and barriers.

### Perceived Benefits

Spanish-speaking caregivers unanimously agreed that the patient portal provided benefits that made it easier to care for their child. The most common features that caregivers mentioned were having access to their child's appointments, medications, and results ( Table 3 ). Caregivers used the patient portal most frequently to view and make appointments, and this functionality was particularly helpful for patients with greater healthcare needs: “he needs to see many specialists, he has to get many appointments…I wasn't going to be able to memorize all the appointments or know when his appointments were going to be…[the patient portal] helped me with that” (Caregiver 19). Other caregivers highlighted that the patient portal helped them avoid missing appointments: “If I forget an appointment, I check [the patient portal] and then I see I have an appointment” (Caregiver 15).

**Table 3:** Perceived benefits of patient portal use

Theme	Definition	Illustrative quote
Appointments	Relates to caregivers using patient portals to view and make appointments	“He needs to see many specialists, he has to get many appointments…I wasn't going to be able to memorize all the appointments or know when his appointments were going to be, so…they talked to me about [the patient portal]”—Caregiver 19 “I like [the patient portal] because due to the many appointments she has with many specialists, I don't miss any. So, we keep an eye on the girl's appointments. Because it sends me a reminder, and I log into the [patient portal]”—Caregiver 14
Medications	Relates to caregivers using patient portals to view current medications	“Sometimes it indicates different dosages, so I log into [patient portal] to check the medication, to make sure I'm not making a mistake with the kind of medication”—Caregiver 6 “The part that shows the medications, because I sometimes have questions about the dosage I should give him, and that [patient portal] shows me the dosages he should take.”—Caregiver 8
Results	Relates to caregivers using patient portals to view laboratory or imaging results	“That's how I used to feel before, because I would say, ‘I want to know what my son's lab results are, I want to look at them by myself,’ right? And now, what they tell me, I can say, ‘That's true, that's how they turned out.’”—Caregiver 17

Caregivers reported benefits with access to their child's medication list. This allowed caregivers to better care for their child by providing medication at the correct dose: “Sometimes it indicates different dosages, so I log into [the patient portal] to check the medication, to make sure I'm not making a mistake” (Caregiver 6). Access to the medication list allowed caregivers to better understand their child's healthcare: “I like to use it, because sometimes…[medications] have a second name, so I like that because it shows the first name and then also the second, and sometimes [doctors] don't understand me…when they ask me about her medications” (Caregiver 9).

Having access to results was a commonly cited benefit. Caregivers reported benefiting from using it while their child was admitted to the hospital: “I like looking at my son's results most. To see how he's doing, whether he needs a transfusion, then I know how his hemoglobin is doing, or his platelets and everything. For me, it has been very helpful, because I can see what my son's lab results are” (Caregiver 17). Seeing results also saved caregiver time and provided reassurance in the outpatient setting: “whenever he's had a blood test done, and I've taken him just to get that done, when I get the results and I can see the sodium levels have gone down, I can be a little bit more at ease without having to call the doctors to find out the results” (Caregiver 17).

These perceived benefits led to greater caregiver understanding of their child's illness, thus empowering them to become more active participants in their child's healthcare. Caregivers shared how they now use the patient portal when they are at clinic visits to provide health information: “I'm with a certain doctor and you're telling your son's medical experience and they ask you questions, I log into [the patient portal] right away and I go, ‘The results for this were this and this and that’” (Caregiver 11).

### Facilitators

Caregivers identified facilitators that made it easier to use patient portals ( Table 4 ). Healthcare professionals (HCPs) were facilitators, as they taught caregivers how to use patient portals more effectively. Multiple types of HCPs were mentioned by the participants, including physicians, nurses, interpreters, and medical assistants: “I've been learning little by little, and sometimes when I go to my appointments, I ask the nurses, ‘You know what? I have a question about [the patient portal]. I’d like to know how this works,' and they've been helping me” (Caregiver 8). HCPs were also important in recommending that caregivers sign up for a patient portal account: “We heard about [patient portal] through the doctors…they said that…we could have more information about the appointments, the medications, and if we had any questions, we could get in touch” (Caregiver 20).

**Table 4:** Facilitators and barriers to patient portal use

Theme	Definition	Illustrative quote
Facilitators		
Healthcare providers	Relates to healthcare providers assisting caregivers in using patient portals more effectively	“I've been learning little by little, and sometimes when I go to my appointments, I ask the nurses, ‘You know what? I have a question about this. I’d like to know how this works,' and they've been helping me.”—Caregiver 8
Barriers		
Language discordance	Relates to differences in caregivers' preferred language and the language used in patient portals, and how that affects the use of the patient portal	“I do notice it's [clinic notes] in English and I only check what it says about how much he weighs, the height and stuff, that's it. I don't check the rest.”—Caregiver 4 “If it were in Spanish, I think I would. For example, I'm checking here right now a thing about some pelvis X-rays for my daughter, and I'm seeing that everything is in English. So I can't know whether anything is dislocated, whether something's not right”—Caregiver 14
Health literacy	Relates to caregivers' ability to find, understand, and use health information, and how that affects the use of patient portals	“We understand that there are words used in the medical environment that will be a bit difficult for us to understand. Besides the fact that they are professional words, they're in English, so we don't understand them. There are some slightly complicated words, and… there are some of us parents who find it difficult to be able to understand them…And the language, especially medical words, we find that even more difficult.”—Caregiver 14
Digital health literacy	Relates to caregivers' skills and ability to seek, find, understand, and appraise health information from electronic sources	“Since they had done some tests on him that time, they said I could look at the results there. But to be honest, since I barely know how to use it, I rarely log in because I don't really know how to use it.”—Caregiver 10 “I don't really know how to use the phone and that's why I also can't connect sometimes. And the doctors, my girl's specialists, have to call me on the phone like that, and they call me when I can't take the [virtual appointment]”—Caregiver 4

### Barriers

Caregivers identified individual factors that negatively affected their ability to use patient portals ( Table 4 ). These include the caregiver's preferred language, health literacy, and digital health literacy. All caregivers highlighted that speaking a LOE was an impediment to using patient portals. Caregivers were less likely to use parts of the patient portal that were not in the caregivers' preferred language, particularly clinical notes: “I do notice [clinical notes are] in English and I only check what it says about how much he weighs, the height and stuff, that's it. I don't check the rest” (Caregiver 19).

A caregiver's health literacy level also affected their ability to use patient portals, as the use of medical jargon made it difficult to understand what was being communicated. This health literacy barrier was compounded by speaking a LOE: “We understand that there are words used in the medical environment that will be a bit difficult for us to understand. Besides the fact that they are professional words, they're in English, so we don't understand them. There are some slightly complicated words…there are some of us parents who find it difficult to be able to understand them” (Caregiver 14).

An individual's digital health literacy affected their ability to use patient portals. 27 Some caregivers highlighted that their lack of understanding of how to navigate the patient portal made it more difficult to access some of its functionalities: “Yes, I can make video calls, but sending—I don't know how to do the rest. How to send messages or download, let's say, any medical reports that I might need, I have to go all the way to the hospital” (Caregiver 16).

## Discussion

Our study reveals that Spanish-speaking caregivers have both similar and distinct experiences using patient portals compared with their English-speaking counterparts. While they find benefits to using these portals, they report unique facilitators and barriers that are influenced by factors including preferred language, digital health literacy, and health literacy. To our knowledge, this is the first study to specifically gather LOE caregiver perspectives on the use of patient portals, and these findings have important and timely implications for health systems promoting language-equitable care as patient portals become ubiquitous.

The Technology Acceptance Model posits that two key factors—perceived usefulness and perceived ease of use—influence one's decision to adopt and use a technology. 25 In our study, participants unanimously recognized the benefits of patient portals, thereby enhancing their perceived usefulness and promoting usage. Conversely, the barriers identified in this study negatively affected perceptions of the portals' ease of use. One's digital health literacy, for example, plays a crucial role in shaping their ease of use. Participants with lower digital health literacy likely perceived patient portals to be challenging to navigate, resulting in decreased actual usage. Importantly, participants in our study exhibited a range of digital health literacy scores, ranging from 5 to 12 on the Digital Health Literacy Questionnaire, highlighting the heterogeneity within the group.

Our participants' experience using patient portals differs from previously published literature on pediatric patient portal use. 30 All caregivers in this study primarily used a smartphone application to access the patient portal, which supports previous research showing that Latinx individuals are more reliant on smartphones for online access compared with white individuals. 31 32 Participants most frequently used the patient portal to access and manage their child's appointments, medications, and results. While English-speaking caregivers also use those functions, they also commonly use the patient portals to read clinical notes and message their providers. 30 33 These differences are likely related to the fact that appointments, medications, and results are already translated or require minimal translation compared with clinical notes and other unstructured data. This disparity in use underscores the need to engage diverse groups of end-users, particularly those who face barriers, to optimize patient portals for varying populations and uses.

Research from the OpenNotes movement highlights the importance of sharing clinical notes, with reported benefits including empowering caregivers through health participation, improved medication adherence, and improved patient safety. 3 4 34 The benefit of shared clinic notes may be even more significant for LOE patients, as they may attend clinic visits with a reduced sense of control and understanding of their provider's communication. 4 Unfortunately, many LOE caregivers underutilize shared clinical notes due to a lack of translation, thereby preventing them from the potential benefits elucidated in the literature. This may lead to intervention-generated inequalities by disproportionately benefiting advantaged groups and thereby increasing the language inequities. This study contributes to ongoing literature that patients and caregivers with LOE experience disparate access and use of digital health tools, including patient portals, which may be exacerbated by other factors such as differences in digital and health literacy. 23 35

While the 21st Century Cures Act created a framework for allowing patient and caregivers to access their medical records, it did not require that health information be provided in patients' preferred language. 36 Nonetheless, the participants in this study report that language discordance is a barrier to optimal patient portal use. Health systems have conventionally relied solely on human translators; machine translation tools, such as Google Translate, have had limited uptake due to their variable accuracy at translating medical text across a range of langugages. 37 However, as artificial intelligence tools such as large language models (LLMs) increase their natural language processing capabilities, these may become effective tools for providing accurate machine translation and promoting language-concordant care. Preliminary studies on the use of ChatGPT (OpenAI, San Francisco, United States), one of many LLMs, suggest that it may have similar performance at translating healthcare materials compared with Google Translate and human translators, with the additional benefit of being able to simplify complex health information into plain language. 38 39 40 In the future, LLMs could be used as a “first-pass” for translation, with human translators editing LLM-generated text to ensure accuracy.

## Limitations

There are several limitations to this study. The participants in our study were already enrolled with patient portals and used them frequently, suggesting that they may have higher digital health literacy and greater access to technology compared with other Spanish-speaking caregivers. Thus, their perspective may be biased toward more positive views of patient portals. Research indicates that social desirability bias may be more prevalent in Latinx and Spanish-speaking caregivers, so there may have been a tendency to report in a way that is perceived as socially acceptable, but may not represent their true experience. 41 While we attempted to reduce this bias by reviewing and piloting our interview guide with Latinx caregivers, social desirability bias may have contributed to reporting positive views of patient portals. Our qualitative study recruited caregivers within a single health system, so our findings may not be fully generalizable to other settings; local institutional differences, such as variability in the implementation and functions of patient portals, may influence caregivers' perceptions of barriers and facilitators. Our study included Spanish-speaking caregivers of patients with chronic conditions, so results may not be generalizable to caregivers who speak other languages or those who have children without chronic conditions.

## Future Direction

Future strategies should adopt a multi-layered approach to promote effective and equitable patient portal use. New technologies should be developed with input from end-users, particularly those who face the greatest challenges in accessing and using patient portals, as they may have unique barriers that should be overcome to ensure equitable patient portal deployment. Operationally, health systems should equip multiple types of HCPs to support patients and caregivers with patient portal queries, including medical assistants, nurses, and clinicians. Consideration should be given to defining which essential data within patient portals should be available in patients' preferred language, so the focus can be on the translation of this data first. LLMs are an exciting potential tool for machine translation, but rigorous evaluation is required to ensure accuracy and safety. Future research should be inclusive of other languages spoken and occur across different health systems to increase generalizability.

## Conclusion

This study highlights the unique experiences that Spanish-speaking caregivers encounter when using patient portals. While patient portals are unequivocally beneficial for managing their child's healthcare, language barriers and differences in digital and health literacy remain significant challenges for caregivers. The study underscores the need for health systems to provide language concordance within patient portals and consider innovative solutions, like incorporating machine translation, to meet the translation needs of patients and caregivers. By addressing these barriers, health systems can promote equitable access and healthcare delivery for Spanish-speaking patients and caregivers.

## Clinical Relevance Statement

Spanish-speaking caregivers unanimously benefit from using patient portals, but barriers, including language discordance, digital health literacy differences, and limited health literacy, prevent them from experiencing the full benefit of patient portals. Health systems should target interventions that specifically address these barriers, with the goal of promoting digital health equity.

## Multiple-Choice Questions

1. What did Spanish-speaking caregivers identify as a facilitator that made it easier to use the patient portal?
  - Online patient portal tutorials.
  - Friends and family members.
  - Healthcare professionals.
  - Community resources.
  **Correct Answer:** The correct answer is option c. Healthcare professionals. Healthcare professionals were identified as a facilitator that made it easier for Spanish-speaking caregivers to use the patient portal. Multiple types of healthcare professionals were identified, including medical assistants, nurses, and doctors. Healthcare professionals encouraged caregivers to sign up initially, and they taught them how to navigate and use the patient portal.
2. What was commonly identified as a patient portal feature that provided benefit for Spanish-speaking caregivers?
  - Viewing appointments.
  - Reading clinic notes.
  - Renewing prescriptions.
  - Accessing general medical content.
  **Correct Answer:** The correct answer is option a. Viewing appointments. Spanish-speaking caregivers frequently used the patient portal to view their child's appointment. Features that required greater English proficiency or were not translated into their preferred language (e.g., clinic notes) were underutilized by Spanish-speaking caregivers.
