# Supplementary material for: Perspectives of Spanish-Speaking Caregivers on Pediatric Patient Portal Use
Source: Appl Clin Inform. 2025 Oct 1;16(4):1244–51. doi: 10.1055/a-2688-3992 (PMC12488237; doi:10.1055/a-2688-3992)
Supplement: Supplementary file 1 — Supplementary Material [file 10-1055-a-2688-3992_27152535.pdf]

Supplementary Appendix A: Digital Health Care Literacy Questionnaire[27]

Q1: I can use applications/programs (like Zoom) on my cell phone, computer, or another electronic device on my own (without asking for help from someone else).

Q2: I can set up a video chat using my cell phone, computer, or another electronic device on my own (without asking for help from someone else).

Q3: I can solve or figure out how to solve basic technical issues on my own (without asking for help from someone else).

Response options:

- Strongly disagree = 0 points
- Disagree = 1 point
- Neutral = 2 points
- Agree = 3 points
- Strongly agree = 4 points

Sum score of individual items to create total score. A higher score indicated higher digital health literacy, with a maximum score of 12.
